# Acupuncture for corona virus disease 2019: A protocol for systematic review and meta analysis

**DOI:** 10.1097/MD.0000000000022231

**Published:** 2020-10-02

**Authors:** Yong Chen, Chengcheng Zhu, Zhangmeng Xu, Yang Song, Hong Zhang

**Affiliations:** a Department of Emergency; b Department of Galactophore, Hospital of Chengdu University of Traditional Chinese Medicine; c Department of Sport Medicine, Chengdu Sport University, Chengdu, Sichuan Province, China.

**Keywords:** acupuncture, corona virus disease 2019, systematic review

## Abstract

**Background:**

There is a worldwide outbreak of coronavirus disease 2019 (COVID-19), at present, accumulative attention has been paid to COVID-19 due to its global prevalence. Acupuncture may play a beneficial role in patients who suffer from COVID-19. In China and East Asia, acupuncture has been widely used to treat diverse diseases for thousands of years, as an important method of treatment now, it plays an indispensable role in the treatment of respiratory diseases in China. This study is designed to determine the efficacy and safety of acupuncture in COVID-19.

**Methods:**

We will search the following sources for the Randomized controlled trials (RCT): The Cochrane Library, PubMed, EMBASE, Web of Science, Chinese Biomedical Literature Database (CBM), Chinese National Knowledge Infrastructure Database (CNKI), Chinese Science, and the Wanfang Database. All the above databases will be searched from the available date of inception until the latest issue. No language or publication restriction will be used. Primary outcomes will include chest CT and nucleic acid detection of respiratory samples.

**Results:**

The results will provide a high-quality synthesis of current evidence for researchers in this subject area.

**Conclusion:**

The conclusion of our study will provide evidence to evaluate whether acupuncture is an effective treatments for patients suffering from COVID-19.

**PROSPERO registration number:**

CRD42020180875.

## 1 Introduction

Since December 2019, a series of unexplained pneumonia cases have been reported in Wuhan, the capital of Hubei, China. On January 12, 2020, now known as severe acute respiratory syndrome coronavirus 2 (SARS-CoV-2) (2019). The etiological agent of COVID-19 has been confirmed as SARS-CoV-2, which is most likely originated from zoonotic coronaviruses, like SARS-CoV, which emerged in 2002.[^1^,^2^] The World Health Organization (WHO) temporarily named this new virus as the 2019 novel coronavirus (2019-nCoV). On February 11, 2020, the WHO officially named the disease caused by the 2019-nCoV as coronavirus disease (COVID-19) and declared that the epidemic is a public health emergency of international concern on January 31, 2020. The COVID-19 epidemic is spreading all over the world, especially in China. COVID-19 has spread to 46 countries internationally, causing over 2 million cases and over 137 thousand of death.[^3^,^4^] Total fatality rate of COVID-19 is estimated at 3.46% by far based on published data from the Chinese Center for Disease Control and Prevention (China CDC).

Coronoviruses have been reported as causes of mild and moderate respiratory infections forover 50 years. Though the outbreak of the Coronovirus is likely to emerge with a zoonotic epidemic related to seafood market, where also has live wildlife trade, bats are accepted major natural reservoir of coronaviruses,[^5^,^6^] it becomes evidently that the virus can spread through human-to-human.^[7]^

COVID-19 is similar to severe acute respiratory syndrome coronavirus (SARS-CoV) virus in its pathogenicity, clinical spectrum, and epidemiology. Comparison of the genome sequences of COVID-19, SARS-CoV, and Middle East Respiratory Syndrome coronavirus (MERS-CoV) showed that COVID-19 has a better sequence identity with SARS-CoV compared with MERS-CoV.^[8]^ The coronavirus is a kind of positive-chain single-stranded RNA virus with a diameter of 80 to 120 nm, which can be classified as a, b, d, and g type. Coronavirus has the characteristics of various strains, wide distribution, and cross species. The COVID-19 belongs to the b genus and has a capsule on which mushroom-like protein spike make the virus crown-like, round or oval size, often pleomorphic, and with a diameter of 60 to 140 nm.^[9]^ Bats are the most important natural hosts. About 35% of the viruses they carry are coronaviruses, from which at least a dozen different coronaviruses have been identified so far.^[10]^ Although several animals have been speculated to be a reservoir for COVID-19, no animal reservoir has been already confirmed. Novel coronavirus causes COVID-19 disease that has similar symptoms as SARS-CoV. Studies suggest that the human receptor for COVID-19 may be angiotensin-converting enzyme 2 (ACE2) receptor similar to that of SARS-CoV. The nucleocapsid (N) protein of COVID-19 has nearly 90% amino acid sequence identity with SARS-CoV. The N protein antibodies of SARS-CoV may cross react with COVID-19 but may not provide cross-immunity. In a similar fashion to SARS-CoV, the N protein of COVID-19 may play an important role in suppressing the RNA interference (RNAi) to overcome the host defense.^[8]^

The most convincing mode of transmission of COVID-19 is inhalation of infectious aerosols,^[11]^ and the main symptoms of the virus are a dry cough, fever, and progressive dyspnea.^[12]^ Also it leads to a serious lung inflammation, acute respiratory distress syndrome (ARDS), cardiac and renal injury, especially in patients with older age, and comorbidities (diabetes mellitus, hypertension, and heart failure).^[13]^ The incubation period is approximately 3 to 14 days. COVID-19 may cause disease ranging from asymptomatic to fatal disease. In elderly patients, COVID-19 infects the lower respiratory tract with the potential of leading to fatal pneumonia.[^14^,^15^,^16^,^17^] In the second week of infection, it progresses to hypoxemia, difficulty in breathing and acute respiratory distress syndrome (ARDS).^[18]^ Patients at this stage may require mechanical ventilation in Intensive Care Unit (ICU) with quarantine facilities. Secondary bacterial infections may set in leading to secondary bacterial pneumonia. The overall rate of deaths per number of diagnosed cases is 4.6%; ranging from 0.2% to 15% according to age group and other health problems.^[19]^

Acupuncture, a main component of Traditional Chinese Medicine (TCM), has been widely adopted to treat respiratory diseases in clinical practice,[^20^,^21^] whose efficacy has been assessed by a number of randomized controlled trials (RTCs).^[22]^ Acupuncture may play a role in the prevention, treatment and rehabilitation of the COVID-19, and relieve the symptoms caused by COVID-19. Acupuncture has been demonstrated to effectively relieving common symptoms in supportive and palliative care, including, anxiety disorders, nausea, insomnia, leukopenia, fatigue as well as vomiting,[^23^,^24^,^25^,^26^,^27^] which might also effectively treat abdominal pain and abdominal distension.[^28^,^29^] Coyle et al^[30]^ have proposed that acupuncture is an effective therapeutic approach for chronic obstructive pulmonary disease (COPD) associated breathlessness. Possible related symptoms of COVID-19 treated with acupuncture include anxiety disorder, insomnia, leucopenia, fatigue, nausea and vomiting, abdominal pain and abdominal distension, breathlessness.^[31]^ The recent systematic review and meta-analysis show that acupuncture can relieve breathlessness in subjects with advanced diseases.^[20]^ Therefore, in this meta-analysis review protocol, our goal is to systematically review the efficacy of acupuncture in relieving the symptoms of discomfort, subsequently improving the physiological function and quality of life of patients with COVID-19 combined with dyspnea.

## 2 Methods

### 2.1 Study registration

The systematic review protocol has been registered in PROSPERO. The registration number: CRD42020180875, the consent of this protocol report is based on the Preferred Reporting Items for Systematic Reviews and Meta-Analyses Protocols (PRISMAP) statement guidelines.

### 2.2 Inclusion criteria for study selection

#### 2.2.1 Type of study

We will include articles related to acupuncture therapy of patients suffering from COVID-19. Due to language restrictions, we will search for articles in English and Chinese in order to get a more objective and true evaluation, all articles included are randomized controlled trial (RCT) type articles.

#### 2.2.2 Type of participant

All patients suffering from COVID-19 will be included regardless of sex, age, race, education, and economic status. Pregnant women, postoperative infections, psychopaths, patients with severe cardiovascular and/or liver, and/or kidney diseases will not be included.

#### 2.2.3 Type of intervention

Acupuncture must be performed in the treatment group, may combined with other treatments, including routine therapy and so on.^[32]^ Patients in the control group will receive other therapeutic approaches other than acupuncture, including routine therapy, placebo, etc.

#### 2.2.4 Type of outcome measure

Primary outcomes: The influence of acupuncture on chest CT and nucleic acid detection of respiratory samples. Secondary outcomes: Accompanying symptoms (such as myalgia, expectoration, stuffiness, runny nose, pharyngalgia, anhelation, chest distress, dyspnea, crackles, headache, nausea, vomiting, anorexia, diarrhea).

### 2.3 Data sources

The following electronic databases will be searched from inception to June 2020: The Cochrane Library, PubMed, EMBASE, Web of Science, Chinese Biomedical Literature Database (CBM), Chinese National Knowledge Infrastructure Database (CNKI), Chinese Science and the Wanfang Database. About other sources, we also plan to manually search for the unpublished conference articles and the bibliography of established publications.

### 2.4 Search strategy

The search terms on PubMed are as follows: acupuncture (e.g., “acupoints” or “dermal needle” or “electroacupuncture”); COVID-19 (e.g., “Corona Virus Disease 2019” or “Corona Virus” or “Novel coronavirus”); randomized controlled trial (e.g., “randomized” or “randomly” or “clinical trial” or “Randomized controlled trial” or “Controlled clinical trial” or “Clinical trials as topic”). Combinations of Medical Subject Headings (MeSH) and text words will be used. The same search term is used in electronic databases in China. These search terms are shown in Table 1.

**Table 1:**
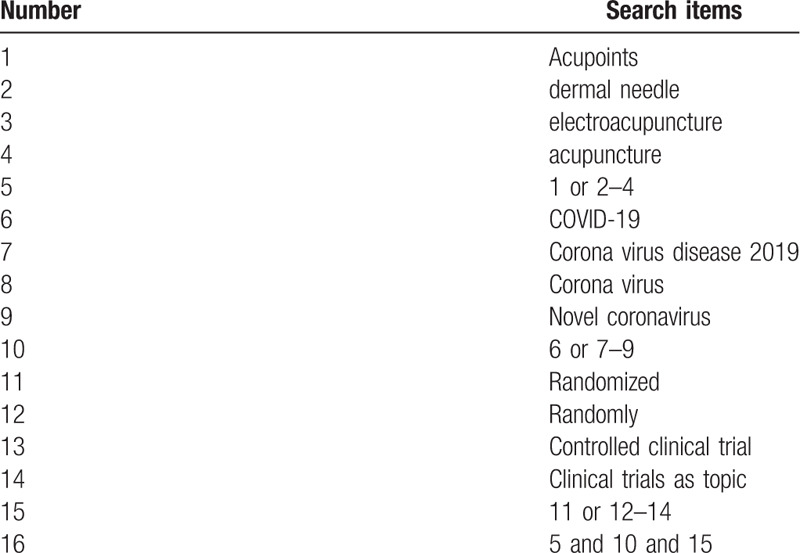
Search strategy for the PubMed database.

### 2.5 Data collection and analysis

#### 2.5.1 Selection of studies

**Figure 1:**
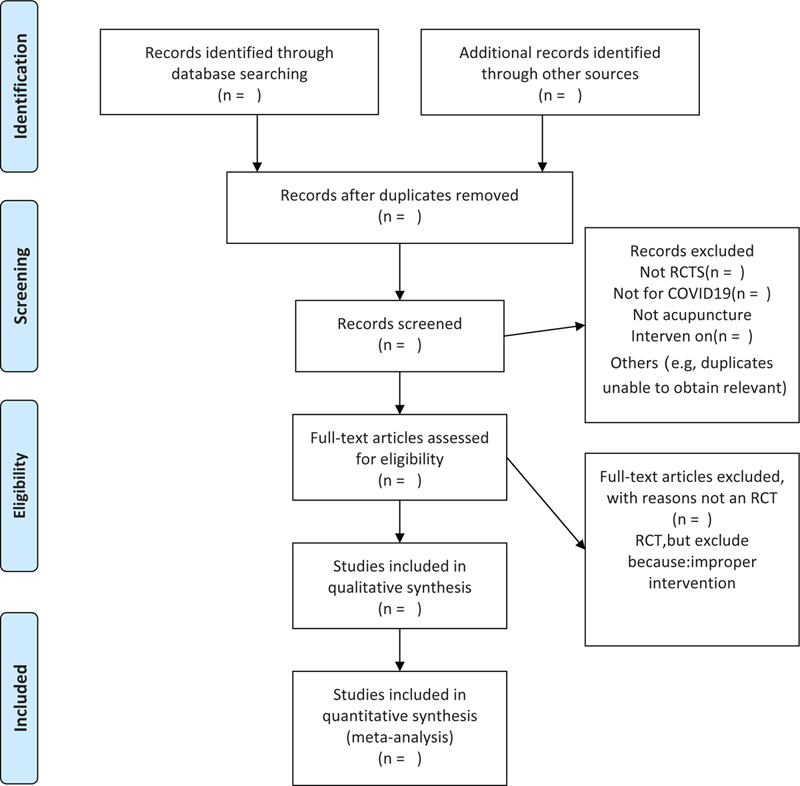
Flow chart of the study.

We chose the PRISMA flow chart to show the process of selecting literature for the entire study (Fig. 1). Before searching the literature, all reviewers will discuss and determine the screening criteria. After the screening requirements are clearly defined, the 2 reviewers will independently review and screen the literature. They screened the titles and abstracts of the literature, in order to get qualified studies, and then excluded some duplicate studies or studies with incomplete information. We will also try to obtain the full text, and the obtained literature will be managed by using EndNote software V.X8 (United States). Any inconsistency is resolved by discussing with the third investigator.

#### 2.5.2 Data extraction and management

The authors will strictly follow the inclusion criteria and select RCT articles related to the topic. Through the analysis of the article, we know participants’ characteristics (height, weight, sex), interventions, outcomes, the study characteristics (press, nationality, journals, research design), adverse reactions, etc. If there is any disagreement between the 2 authors in the literature data extraction, the third article participant will discuss the decision together. If there is a lack of data in the literature, we will contact the author or publisher as much as possible.

#### 2.5.3 Assessment of risk of bias in included studies

We will use the Cochrane collaborative tool to independently assess the risk of bias in the included studies. We will evaluate the following aspects of the article: sequence generation, assignment sequence hiding, blindness of participants and staff, outcome evaluators, incomplete result data, selective result reporting, and other sources of bias. The risk of bias is evaluated at 3 levels, namely, low risk, high risk, and ambiguity. If the information is vague, we will try to contact the author of the article.

#### 2.5.4 Measures of treatment effect

In this protocol, we will use 95% confidence interval (CI) risk ratio (RR) to rigorously analyze the dichotomous data. And for the continuous data, mean difference (MD) or standard MD (SMD) is used to measure the efficacy of 95% CI.

#### 2.5.5 Unit of analysis issues

We will include data from parallel group design studies for meta-analysis. In these trials, we will collect and analyze individual measurements of each outcome for each participant.

#### 2.5.6 Management of missing data

We will try our best to ensure the integrity of the data. If the included RCT data are not complete, we will try every means to contact the corresponding author of the article, including sending emails or making a phone call. If the corresponding author cannot be contacted, we will remove the experiment with incomplete data. After data integrity is assured, intention analysis therapy and sensitivity analysis will be performed.

#### 2.5.7 Assessment of heterogeneity

For the detection of heterogeneity, the *I* ^2^ test will be used to detect the heterogeneity among trials. When the *I* ^2^ test value is <50% and *P* value >1, we think there is no heterogeneity between these trials, and when the *I* ^2^ test value is >50% and the *P* value is <1, there is significant heterogeneity between these included trials. If significant differences are detected, we will analyze the possible causes of heterogeneity, and then we can use the random-effects model.

#### 2.5.8 Assessment of reporting biases

In this analysis, once >10 trials are included, funnel plots could be used to test for reporting bias.

#### 2.5.9 Data synthesis

We will use Review Manager Software (RevMan) V.5.3 (Copenhagen, Denmark) for data analysis and quantitative data synthesis. If there is no finding of statistical heterogeneity, the fixed-effect model is used for data synthesis. If there is significant statistical heterogeneity, we will use the random effect model, and all participants will explore the possible causes from a clinical and methodological perspective and provide a descriptive or subgroup analysis.

#### 2.5.10 Subgroup analysis

Subgroup analysis will be performed to explain heterogeneity if possible. Factors such as different types of control interventions and different outcomes will be considered.

#### 2.5.11 Sensitivity analysis

Based on sample size, study design, heterogeneous quality, methodological quality, and statistical model, sensitivity analysis will be performed to exclude trials with quality defects and ensure the stability of the analysis results.

#### 2.5.12 Grading the quality of evidence

This paper will use the evidence quality rating method to evaluate the results obtained form this analysis. GRADE is generally applied to a large amount of evidence. It has 4 evaluation levels, namely, high, medium, low, and very low. GRADE was used to evaluate the bias, inconsistencies, discontinuities, and inaccuracies of test results. In the context of the system review, quality reflects our confidence in the effectiveness of assessment.^[33]^

#### 2.5.13 Ethical review and informed consent of patients

Ethics and dissemination: The content of this article does not involve moral approval or ethical review and will be presented in print or at relevant conferences.

## 3 Discussion

Like the outbreaks caused by SARS and MERS, the recent outbreak of COVID-19 in China is creating a substantial public health challenge. The pathogenesis and clinical symptoms related to severe respiratory disease were described many years ago in TCM classics.^[34]^ Currently there are many studies on application of TCM in COVID-19, such as the clinical outcome, pathogenesis, and the current application of TCM on COVID-19.[^34^,^35^] The effective treatment of COVID-19 is of great significance. Acupuncture could help make up for the deficiency of current treatment of COVID-19,^[36]^ which is worth studying. Summarize the available evidence of the treatment of COVID-19 with acupuncture, and evaluate the efficacy and the adverse effects of these treatments. Our findings may help clinicians and health professionals to make clinical decisions about the treatment of patients with COVID-19 for further study in the future.

The strength of our review includes the following 3 points. First, it is the first systematic review concerning the safety and effectiveness of acupuncture in COVID-19. Second, only RCTs are included in our systematic review, which are more likely to provide unbiased information than other study designs. Third, the comprehensive search strategy renders in depth searching lists as well as trial registries associated with acupuncture and COVID-19. This protocol may have the limitation that we developed it based on designs of current registered clinical trials, but we will update our proposed methods promptly on our PROSPERO record if these methods change. This review will help explore the potential role for acupuncture in the treatment or prevention of viral infections.

## Author contributions

**Conceptualization:** Yong Chen.

**Data curation:** Chengcheng Zhu, Zhangmeng Xu, Yang Song.

**Formal analysis:** Zhangmeng Xu, Chengcheng Zhu, Yang Song.

**Resources:** Hong Zhang.

**Software:** Zhangmeng Xu.

**Writing – original draft:** Yong Chen.

**Writing – review & editing:** Chengcheng Zhu, Zhangmeng Xu, Hong Zhang.
